# Development and initial validation of the Morningness-Eveningness Exercise Preference Questionnaire (MEEPQ) in Japanese university students

**DOI:** 10.1371/journal.pone.0200870

**Published:** 2018-07-18

**Authors:** Ryo Miyazaki, Hitoshi Ando, Tomoko Hamasaki, Yukito Higuchi, Kazushige Oshita, Tomoki Tashiro, Naoki Sakane

**Affiliations:** 1 Faculty of Human Sciences, Shimane University, Shimane, Japan; 2 Department of Sports and Health Sciences, Faculty of Human Sciences, University of East Asia, Yamaguchi, Japan; 3 Department of Cellular and Molecular Function Analysis, Kanazawa University Graduate School of Medical Sciences, Ishikawa, Japan; 4 Department of Nutrition Faculty of Home Economics, Kyushu Women's University, Fukuoka, Japan; 5 Department of Sports Science, Kyushu Kyoritsu University, Fukuoka, Japan; 6 Division of Preventive Medicine, Clinical Research Institute for Endocrine and Metabolic Disease, National Hospital Organization, Kyoto Medical Center, Kyoto, Japan; University of Maiduguri College of Medical Sciences, NIGERIA

## Abstract

The aim of this study was to develop a questionnaire to conveniently assess the diurnal preferences of physical activity (PA) in Japanese university students. A total of 219 subjects completed our novel Morningness-eveningness Exercise Preference Questionnaire (MEEPQ). The MEEPQ consisted of 30 items (15 items for the morning and the same 15 items for the evening) rated on a 5-point Likert scale concerning their preference for participating in PA in the morning and evening. The morning score (MS) and evening score (ES) were determined by summing each of the respective 15 items. The internal consistency and construct validity were assessed, and a factor analysis was conducted. To examine the external validity of the MEEPQ, participants wore an accelerometer for seven consecutive days to measure their PA levels objectively. Finally, the test-retest reliability was evaluated at a one-month interval. The MEEPQ showed excellent internal consistency (Cronbach's alpha = 0.896) and construct validity (morning KMO = 0.913, evening KMO = 0.875). A factor analysis showed a three-factor structure involving Physical Wellness (MEEPQ-W), Psychological Well-Being (MEEPQ-P) and Exercise Barrier (MEEPQ-B). The percent of variance was largest for MEEPQ-W in the morning (45.2%) and MEEPQ-P in the evening (40.8%). Test-retest showed that MEEPQ scores had fair repeatability. Significant and positive associations between scores and objectively measured PA levels were found in the MS and 6–9 AM PA and in the ES and 6–9 PM and 9 PM– 0 AM PA (all p<0.05). In summary, the novel MEEPQ showed relatively good agreement and thus can be used for Japanese university student samples. In the MEEPQ, three factors (the physical wellness, psychological well-being and exercise barrier) contributed to a morning or evening PA preference. The summed scores were significantly associated with the objectively measured PA levels in both the morning and evening. Therefore the MEEPQ appears to be a suitable tool for assessing diurnal PA preferences.

## Introduction

Physical activity (PA) confers a range of health benefits, including a reduced risk of developing coronary heart disease, diabetes and mental illness [1]. Unfortunately, few individuals undertake PA levels necessary to realize these benefits. Population-level surveys in Japan have shown a long-term decrease over time in the daily PA levels for 15- to 19-year-olds [2]. Despite several decades of effort, new and more effective methods for increasing PA levels are warranted.

One potential approach for addressing the low levels of PA and establishing PA habits is to consider individual lifestyles. One critical determinant of lifestyles is the human circadian function. Morningness-eveningness, also called the “chronotype” is the most widely studied characteristic of human circadian functioning, referring to the individual differences in the circadian phase position and preferred hours of activity [3]. The morningness-eveningness is determined by various factors, such as the rise and bed time, season, age and gender. Although factors that may modify PA levels have been studied, little is known about the morningness-eveningness of PA.

In fact, diurnal PA rhythms in humans have been shown to be quite diverse in previous studies. For instance, while elite individual sport endurance athletes, especially those who are required to attend morning training, such as triathletes and cyclists, are more often early risers than control populations, the chronotype distribution in team sport players is similar to that of the general population [4]. Another study observed that the circadian rhythm of objectively measured PA was influenced by the chronotype [5]. In their study, evening-oriented university students showed an acrophase of PA more than 2 h later than that of morning-oriented counterparts [5]. These findings suggested that we should recognize the importance to morningness-eveningness of PA (i.e. the ‘preference of PA timing in a day’).

Few studies have reported on preferences in diurnal PA rhythms. South African researchers have reported that, even though athletes more often preferred to practice in the morning than the normal young population, the time of day at which the athletes preferred to train was significantly associated with their chronotype [6]. This finding clearly showed that individuals have their own preferred timing for engaging in PA. Supporting this, a previous study comparing morning and evening time-trial performance showed that swimmers showed better performances and felt less fatigue when they engaged in PA at their usual time of day (morning or evening) [7]. In their study, morning-/evening-type swimmers or those who trained in the morning/evening swam the fastest in the corresponding time of day [7]. Recently, two important articles examining diurnal PA preferences have been published. Schondube et al. showed that, among normal university students, positive affective valance (such as joy) was a significant predictor of exercise participation on a day level [8]. Hisler et al. revealed that diurnal preference predicted both self-reported exercise and the objectively measured exercise frequency [9]. These findings show that understanding one’s personal mornigness-eveningness of PA preference can improve the adherence of PA and PA performance, in elite athletes as well as in normal young people. However, to our knowledge, no tool for conveniently assessing diurnal PA preference has yet been developed.

Morningness-eveningness is often evaluated using self-rated questionnaires. The assessment of diurnal preference or the preferred timing of sleep and activity is generally based on comprehensive questionnaires, such as the Horne-Östberg Morningness-Eveningness Questionnaire (MEQ) [10], the Munich ChronoType Questionnaire (MCTQ) [11] and the Composite Scale of Morningness (CSM) [12]. Although these scales have been validated against biological variables and sleep behavior of morning- and evening-type participants, these scales (and the subsequent improved version of these scales) were developed to assess ‘overall morningness-eveningness’ and cannot distinguish specific behavioral preferences such as eating, sleep or PA. Concerning eating habits, the Night Eating Questionnaire (NEQ) [13] has been validated in adults. However, to our knowledge, there is no tool for directly assessing the morningness-eveningness of PA preference. Because individuals’ differences in PA behavior during the day are thought to be more diverse than differences in eating or sleeping habits, developing a tool to assess an individual’s diurnal PA preference would provide valuable information for individuals engaging in PA.

Therefore, the aim of this study was to develop a convenient, self-administrated questionnaire to assess the diurnal preferences of PA among university students and examine the validity of the questionnaire.

## Methods

### Participants and survey procedures

This cross-sectional study was based on a self-administered questionnaire survey among male and female university students (n = 219; 121 males and 98 females; aged 20.4±1.1 years) from three universities in Yamaguchi and Fukuoka Prefectures in Japan. Male participants were recruited from two sports-related universities, and female participants were recruited from a nutrition-related university. All subjects were free of any severe medical history that affected their PA habits and were not taking any medications.

For questionnaire development and the exploratory analysis, 216 university students (120 males and 96 females; aged 20.2±1.1 years; the mean body mass index [BMI], calculated from the self-reported weight and height, was 22.5±3.3 kg/m^2^) completed our novel Morningness-eveningness Exercise Preference Questionnaire (MEEPQ). To assess the reliability of the MEEPQ, the university students completed the MEEPQ twice with one-month intervals. In addition, we asked the participants about typical rise and bed times.

To collect the data, researchers distributed the MEEPQ questionnaire to students during lectures. Missing answers or logistical errors were checked by staff, and when necessary, the student was asked to complete the questionnaire again. Surveys were completed from February to June 2016. The study protocol was reviewed and approved by the Ethics Committee of the University of East Asia (Yamaguchi, Japan) (2015–12) and was in accordance with the principles of the Declaration of Helsinki. All subjects provided their written informed consent.

### Questionnaire development

#### The novel questionnaire MEEPQ

To assess the diurnal PA preference, especially in the morning and evening, we defined the morning hours at 6–9 AM and the evening hours at 3–6 PM, based on the definition of the Japan Meteorological Agency (http://www.jma.go.jp/jma/kishou/know/yougo_hp/saibun.html). To assess the morningness-eveningness of PA preferences, we developed our MEEPQ with 30 items, including 15 each for morning and evening. The wording of the 15 items was the same for both section except for ‘morning’ and ‘evening’. Each question could be answered using a 5-point Likert scale as follows: 1 = strongly disagree, 2 = disagree, 3 = neither disagree/agree, 4 = agree, 5 = strongly agree. A higher total score for a respondent indicated a higher preference of morning/evening PA. The questionnaire was developed by experts (one physician, two health fitness programmers, one handball coach, one registered dietitian, one public health scientist and one chronopharmacology scientist). An example of the questionnaire is shown in Tables 1 and 2.

**Table 1:** Original MEEPQ-morning items and percentage of informants selecting each option. If you engaged in physical activity in the morning (6–9 AM), what kind of advantages or disadvantages would you experience? For each question, select the answer choice that best describes you.

No.	Item	1. Strongly disagree	2. disagree	3. neither disagree/agree	4. agree	5. strongly agree	score
1	It is fun to do PA in the morning.	13.9	20.4	23.6	29.6	12.5	3.1 ±1.2
2	PA in the morning relieves stress.	11.6	14.8	26.4	32.9	14.4	3.2 ±1.2
3	It is refreshing to engage in PA in the morning.	7.4	12.0	22.7	37.5	20.4	3.5 ±1.2
4	Engaging in PA in the morning gives me a sense of fulfillment and achievement.	9.7	10.6	24.5	34.3	20.8	3.5 ±1.2
5	PA in the morning is good for maintaining physical fitness.	10.2	11.1	27.3	36.6	14.8	3.3 ±1.2
6	PA in the morning allows me to wake up feeling good.	6.9	13.4	20.4	38.0	21.3	3.5 ±1.2
7	PA in the morning is useful for preventing and improving diseases.	4.2	6.5	24.1	45.4	19.9	3.7 ±1.0
8	Morning PA helps with weight management.	5.6	6.9	27.8	39.4	20.4	3.6 ±1.1
9	Engaging in PA in the morning allows me to get a good night’s sleep.	10.6	12.5	33.8	29.2	13.9	3.2 ±1.2
10	PA in the morning makes me exhausted.	3.2	10.6	21.8	38.0	26.4	3.7 ±1.1
11	PA in the morning allows me to deepen friendships.	14.8	19.9	48.6	11.6	5.1	2.7 ±1.0
12	PA in the morning may hinder work and housework.	6.9	20.4	36.1	28.2	8.3	3.1 ±1.0
13	It is difficult to make time to engage in PA in the morning.	5.1	17.6	17.1	25.9	34.3	3.1 ±1.3
14	If I engage in PA in the morning, there is a danger of getting involved in crime and accidents.	31.0	28.2	35.2	4.2	1.4	3.1 ±1.0
15	Generally speaking, PA in the morning is helpful for improving my health.	4.2	5.6	18.5	44.9	26.9	3.8 ±1.0
	Total score (out of 75)						50.0 ±10.2

Data are shown as the percent (%) or mean ±SD. PA, physical activity; SD, standard deviation.

**Table 2:** Original MEEPQ-evening items and percentage of informants selecting each option. If you engaged in physical activity in the evening (3–6 PM), what kind of advantages or disadvantages would you experience? For each question, select the answer choice that best describes you.

No.	Item	1. Strongly disagree	2. disagree	3. neither disagree/agree	4. agree	5. strongly agree	score
1	It is fun to do PA in the evening.	4.2	11.1	18.5	47.7	18.5	3.7 ±1.0
2	PA in the evening relieves stress.	3.7	10.6	20.4	44.9	20.4	3.7 ±1.0
3	It is refreshing to engage in PA in the evening.	4.2	12.0	27.8	39.4	16.7	3.5 ±1.0
4	Engaging in PA in the evening gives me a sense of fulfillment and achievement.	3.2	6.9	25.9	41.7	22.2	3.7 ±1.0
5	PA in the evening is good for maintaining physical fitness.	2.8	6.5	24.1	46.3	20.4	3.8 ±0.9
6	PA in the evening allows me to wake up feeling good.	11.1	21.3	33.3	22.2	12.0	3.0 ±1.2
7	PA in the evening is useful for preventing and improving diseases.	2.3	6.5	27.8	42.6	20.8	3.7 ±0.9
8	evening PA helps with weight management.	1.9	6.5	29.6	40.7	21.3	3.7 ±0.9
9	Engaging in PA in the evening allows me to get a good night’s sleep.	1.9	6.9	17.6	43.5	30.1	3.9 ±1.0
10	PA in the evening makes me exhausted.	1.9	6.9	19.0	46.3	25.9	3.9 ±0.9
11	PA in the evening allows me to deepen friendships.	6.5	8.8	48.1	28.2	8.3	3.2 ±1.0
12	PA in the evening may hinder work and housework.	6.9	22.7	42.1	19.9	8.3	3.0 ±1.0
13	It is difficult to make time to engage in PA in the evening.	6.5	21.8	35.6	24.5	11.6	3.1 ±1.1
14	If I engage in PA in the evening, there is a danger of getting involved in crime and accidents.	15.7	19.4	34.3	24.5	6.0	2.9±1.1
15	Generally speaking, PA in the evening is helpful for improving my health.	2.8	4.6	28.2	42.6	21.8	3.8 ±0.9
	Total score (out of 75)						52.6 ±9.0

Data are shown as the percent (%) or mean ±SD. PA, physical activity; SD, standard deviation.

#### Scoring the MEEPQ

To ensure simplicity of the MEEPQ scoring, scores from all 15 items (morning score [MS] and evening score [ES]) were summed, providing a range from 15 to 75, with higher scores indicating higher preferences for morning or evening PA.

### Validity testing

#### Internal validity

The MEEPQ items were tested for construct validity using Kaiser-Meyer-Olkin (KMO). A KMO value closer to 1 is good, and 0.6 is acceptable. Items with KMO values <0.5 or low communality are usually considered items that can be dropped from the analysis [14]. The reliability of the questionnaire was tested using two methods: an internal consistency test (with a Cronbach’s alpha coefficient) and a stability test (split-half test with a Spearman-Brown coefficient).

#### External validity

To evaluate the external validity, objectively measured PA levels and the MEQ score were used. First, PA levels were objectively measured for at least seven consecutive days using a single-axis electronic accelerometer (Lifecorder Plus; Suzuken, Nagoya, Japan). Participants were instructed to wear the accelerometer throughout the day except during sleep and water-related activities (e.g. bathing, swimming). When they did not wear the accelerometer, we asked them to record the non-wearing time in their lifestyle log.

The Lifecorder is a uniaxial piezoelectric accelerometer that is small and lightweight (72.0 × 42.0 × 29.1 mm, 45 g). The Lifecorder samples vertical acceleration ranging between 0.06 G and 1.94 G at 32 Hz and classifies the response into 1 of 11 intensity levels from 0 to 9 (0, 0.5, 1, 2, 3, 4, 5, 6, 7, 8, 9) every 2 min [15]. Because Kumahara et al. [15] showed that level 1 or higher corresponds to light activity (≥ 1.8 METs), we defined the PA level as activity at level 1 or higher. The PA data were entered into a personal computer by a researcher using Lifestyle Coach 2 version 2.1 software (Suzuken). The validity of the accelerometer measurements was described by Kumahara et al. [15]. The PA level data were expressed as the sum of the weekly PA levels (6–9 AM, 9 AM—0 PM, 0–3 PM, 3–6 PM, 6–9 PM, 9 PM– 0 AM), defined by the Japan Meteorological Agency, as described above.

Second, to compare the MEEPQ with the validated diurnal preference questionnaire, we collected the Japanese version [16] of the MEQ [10], which comprises 19 items and is aimed at measuring several aspects of morningness, including sleep habits, sleepiness, and the preferred time for performing activities that require physical or mental alertness. Fourteen of the items are assessed on a Likert scale format with 4 response choices, while answers to the 5 remaining items are marked along a continuum. The MEQ score ranges from 16–86, with higher scores indicating a higher degree of morningness.

### Factor analyses

An exploratory factor analysis was used to assess the validity of the questionnaire and to identify the items underlying each factor [14]. The factor analysis involved a varimax rotation on the retained factors to help with interpretation. The next analysis involved interpreting the rotated solution by identifying which items were loaded on each retained factor, the conceptual meaning of items that were loaded on the same factor and conceptual differences in items that loaded on different factors. Pattern loadings near ≥0.35 (absolute value) were used to interpret the results [17].

### Reliability testing

Intraclass correlation coefficients (ICCs) with 95% confidence intervals (CIs) were used to assess test-retest reliability by comparing participants’ responses on the self-administered time-specific items at Times 1 and 2. The interval between the test-retest studies was one month. The ICCs were calculated using a two-way mixed model based on absolute agreement. We followed the ratings suggested by Landis and Koch [18] an agreement level: 0–0.2 poor, 0.2–0.4 fair, 0.4–0.6 moderate, 0.6–0.8 substantial, and 0.8-<1.0 almost perfect.

### Statistical analyses

Data are expressed as the mean ± standard deviation (SD), with p < 0.05 considered significant. The correlations between the parameters are provided as Spearman’s rank correlation coefficients. All analyses were conducted using SPSS for Windows Ver. 23.0 (SPSS Inc., Chicago, IL, USA).

## Results

### Participant characteristics

The characteristics of the participants were shown in Table 3. The mean MEQ score was 47.3±8.2, which is comparable to previous Japanese university student samples (mean MEQ score 47.2±8.0) [16].

**Table 3:** Characteristics of participants.

Variables	Mean	SD
Age (years)	20.2	1.1
Sex, n (%) female	96 (44.4%)	
Height (cm)	164.6	8.7
Body weight (kg)	61.6	12.8
BMI (kg/m^2^)	22.5	3.3
MEQ (score) (n = 213)	47.3	8.2
Rise time (hour: min) (n = 215)	7:10	1:10
Bed time (hour: min) (n = 215)	24:36	1:06

n = 216. BMI, body mass index; MEQ, morningness-eveningness questionnaire.

### Validity testing

#### Internal validity

Bartlett’s Test of Sphericity and KMO were as follows: the morning (approximate χ^2^ = 1881.4, df = 105, p < 0.001, and KMO = 0.913) and the evening (approximate χ^2^ = 1671.4, df = 105, p < 0.001, and KMO = 0.875), showing a suitable correlation matrix and sampling adequacy for the factor analysis. The analysis of the internal consistency showed that Cronbach’s alpha was 0.896, and the Spearman-Brown coefficient was 0.747, which suggest high stability and internal consistency of the 15 items in the MEEPQ.

#### External validity

Significant and positive associations between scores and objectively measured PA levels were found in the MS and 6–9 AM PA (r = 0.255, p = 0.008) and in the ES and 6–9 PM PA (r = 0.285, p = 0.003) and 9 PM– 0 AM PA (r = 0.315, p = 0.001) (Table 4). Furthermore, significant and positive associations were noted between MS and MEQ (r = 0.284, p<0.001) and ES and bed time (r = 0.141, p<0.05). However, the remaining parameters were not associated with either MS or ES (Table 5, S1 Fig).

**Table 4:** Relationships between the MEEPQ scores and objectively measured activity levels (weekly mean levels).

		morning score (MS)		evening score (ES)		
		r	p		r	p
activity	hour					
	6–9	0.255	0.008		0.123	0.209
	9–12	0.180	0.065		0.038	0.702
	12–15	0.208	0.032		0.067	0.496
	15–18	0.080	0.417		0.102	0.300
	18–21	0.009	0.924		0.285	0.003
	21–24	-0.117	0.234		0.315	0.001

n = 108. Spearman's coefficient.

**Table 5:** Correlations between the MEEPQ, rise time, bed time and MEQ score.

	morning score (MS)	evening score (ES)	MEQ score	Rise time	Bed time
morning score (MS)	-	0.588**	0.284**	-0.069	0.064
evening score (ES)	0.588**	-	0.118	0.042	0.141*

n = 213 (due to missing data of MEQ). Spearman's coefficient. MEQ, morningness-eveningness questionnaire.

* p<0.05

** p<0.01.

### Factor analysis and interpretation of the factors

#### Rotation of the chosen factors

The rotated factor pattern of pattern loadings from the varimax rotation of three factors is shown in Tables 6 and 7. The pattern loadings in this matrix are essentially standardized regression coefficients comparable to those obtained in multiple regression. Their absolute values reflect the unique contribution that each factor makes to the variance of the observed item. We used this matrix to determine which groups of items are measuring a given factor and to interpret the meaning of each factor.

**Table 6:** Rotated factor pattern (MEEPQ-morning).

	No.	Item	Factor 1	Factor 2	Factor 3
Physical wellness	7	PA in the morning is useful for preventing and improving diseases.	**0.928**	-0.073	0.025
	15	Generally speaking, PA in the morning is helpful for improving my health.	**0.761**	0.046	0.061
	8	Morning PA helps with weight management.	**0.750**	0.067	0.080
	9	Engaging in PA in the morning allows me to get a good night’s sleep.	**0.691**	0.021	-0.106
	5	PA in the morning is good for maintaining physical fitness.	**0.535**	0.293	0.007
	6	PA in the morning allows me to wake up feeling good.	**0.534**	0.251	-0.200
Psychological well-being	3	It is refreshing to engage in PA in the morning.	0.035	**0.881**	0.006
	2	PA in the morning relieves stress.	0.085	**0.837**	0.054
	4	Engaging in PA in the morning gives me a sense of fulfillment and achievement.	0.137	**0.798**	0.089
	1	It is fun to do PA in the morning.	0.048	**0.785**	-0.043
	11	PA in the morning allows me to deepen friendships.	0.269	**0.376**	0.022
Exercise barrier	13	It is difficult to make time to engage in PA in the morning.	-0.036	0.079	**0.653**
	12	PA in the morning may hinder work and housework.	-0.064	0.060	**0.645**
	10	PA in the morning makes me exhausted.	0.159	-0.248	**0.538**
	14	If I engage in PA in the morning, there is a danger of getting involved in crime and accidents.	-0.078	0.171	0.267
	Initial Eigenvalues		6.8	2.0	1.1
	Percent of variance explained		45.2	58.3	65.5
	Cronbach's alpha		0.896	0.913	0.642

**Table 7:** Rotated factor pattern (MEEPQ-evening).

	No.	Item	Factor 1	Factor 2	Factor 3
Physical wellness	8	evening PA helps with weight management.	0.006	**0.905**	-0.154
	7	PA in the evening is useful for preventing and improving diseases.	0.197	**0.663**	0.004
	5	PA in the evening is good for maintaining physical fitness.	0.259	**0.623**	0.004
	15	Generally speaking, PA in the evening is helpful for improving my health.	0.167	**0.604**	0.067
	10	PA in the evening makes me exhausted.	-0.241	**0.554**	0.202
	9	Engaging in PA in the evening allows me to get a good night’s sleep.	0.316	**0.372**	-0.003
Psychological well-being	2	PA in the evening relieves stress.	**0.959**	-0.083	-0.001
	3	It is refreshing to engage in PA in the evening.	**0.904**	-0.032	0.018
	1	It is fun to do PA in the evening.	**0.893**	-0.063	-0.073
	4	Engaging in PA in the evening gives me a sense of fulfillment and achievement.	**0.759**	0.125	0.004
	6	PA in the evening allows me to wake up feeling good.	**0.495**	0.039	0.073
	11	PA in the evening allows me to deepen friendships.	**0.453**	0.080	0.123
Exercise barrier	13	PA in the evening may hinder work and housework.	0.017	-0.016	**0.751**
	12	It is difficult to make time to engage in PA in the evening.	-0.006	0.079	**0.623**
	14	If I engage in PA in the evening, there is a danger of getting involved in crime and accidents.	0.137	-0.055	**0.517**
	Initial Eigenvalues		6.1	2.0	1.1
	Percent of variance explained		40.8	54.0	61.2
	Cronbach's alpha		0.882	0.851	0.654

The exploratory factor analysis uncovered 3 underlying PA factors represented by 15 noncomplex, high-loading (β > 0.35) items that accounted for 65.5% (morning) and 61.2% (evening) of all the variance in the questionnaire data. The three MEEPQ scales were labeled Physical Wellness (MEEPQ-W), Psychological Well-Being (MEEPQ-P) and Exercise Barrier (MEEPQ-B).

#### Interpretation of the factors

Although the orders of factors and the components of each factor that contributed to the variance in the MEEPQ differed between the morning and the evening, the MEEPQ comprised the MEEPQ-W, the MEEPQ-P and the MEEPQ-B. The percent of variance was largest for MEEPQ-W in the morning (45.2%) and MEEPQ-P in the evening (40.8%). MEEPQ-B showed the least contribution, both in the morning and the evening.

### Test-retest reliability

There were 56 participants with retest the MEEPQ data available with interval of one month. The agreement ICC was 0.44 (95% CI 0.21–0.63) in the MS and 0.47 (95% CI 0.25–0.65) in the ES, demonstrating ‘moderate’ reliability, respectively.

## Discussion

The objective of the present study was to develop a convenient questionnaire that assesses the diurnal PA preferences for use in a population of university students in Japan. To our knowledge, this was the first study to assess the morningness-eveningness of PA preference using a convenient tool. We achieved the successful development and initial validation of the MEEPQ, which showed appropriate validity, reliability, simplicity and functionality. The 3-scale, 15-item MEEPQ accounted for 65.5% in the morning PA and 61.2% in the evening PA in the variance in the questionnaire data and demonstrated strong internal consistency. This was accomplished using only 3 constructs and 15 questions. Furthermore, the summed scores (both morning and evening) were significantly related to the objectively measured PA levels in morning (6–9 AM) and evening (6–9 PM and 9 PM-0 AM). The successful development and preliminary validation of the MEEPQ demonstrates the feasibility of using a short, multi-construct questionnaire to explain the morningness-eveningness of PA preferences in young population.

Our exploratory factor analysis revealed three underlying factors within the MEEPQ, relating to a subjective assessment of the physical wellness, psychological well-being and exercise barrier. Interestingly, the components of morningness-eveningness of PA differed between the morning and evening. The largest determinants of the MEEPQ were MEEPQ-W (45.2%) in the morning and MEEPQ-P in the evening (40.8%). These results suggest that PA preferences were basically explained by the physical wellness in the morning and the psychological well-being in the evening. It has been well documented that, regardless of age, physically active (young [19] and middle-aged [20]) individuals are more morning-orientated than their respective control groups when assessed using the MEQ. Consequently, previous cross-sectional studies have shown that morning-oriented middle-aged people had more desirable metabolic profiles than others [20, 21]. Taken together, these findings suggest that morning preference of PA is related to physical health awareness, such as physical wellness. In contrast, in the evening, the largest contributor to PA preference was the psychological well-being. This result suggests that evening PA is associated with one’s overall sense of fulfillment, not only physical issues. In the MEEPQ, MEEPQ-P was interpreted as a complex factor comprising broad indicators, such as reducing stress, achieving good sleep and building or strengthening friendships. Because the largest load in MEEPQ-P in the evening was ‘reducing stress’ (0.959) (Table 7), coping with negative feelings may inform the evening PA preferences.

However, while the associations between evening PA and positive/negative feelings have been described in previous studies, whether or not evening PA assists in coping with stress is unclear. Laborde et al. reported that, among young athletes, overall eveningness was negatively related to a positive psychological status and was unrelated to sport participation [22]. One possible explanation for this discrepancy with our results might be that our study participants were generally non-athletes. Interestingly, another previous study suggested that, regardless of circadian typology or sex, physically active individuals were better at controlling their negative psychological status than sedentary individuals [23]. Our study cannot clarify the causal relationship between the morningness/eveningness of PA and its components (physical wellness and psychological well-being). Further research will be needed to establish the relationship between the morningness/eveningness of PA and these parameters, particularly with regard to the characteristics of evening PA preferences.

MEEPQ-B made the smallest contribution to the MEEPQ in both the morning and evening. These results imply that exercise barriers are a significant factor influencing the morningness/eveningness of PA, even though their contribution is relatively small. This could be partly because this questionnaire includes not only indoor PA but also outdoor PA (Item No.14). Interestingly, the percent of variance in the morning (7.2%) and evening (7.2%) was quite similar. A previous study examined the relationships between walkability of a neighborhood and the hour-by-hour PA levels in Swedish adult populations [24]. They reported that living in a high-walkability neighborhood was associated with more daily moderate-intensity PA than living in a low-walkability neighborhood, mainly in the afternoon/early evening during weekdays, whereas it appeared across the middle of the day (from noon to 4 PM) during weekend days [24]. Jansen et al. analyzed the habitual objectively measured hour-by-hour PA and found that middle-aged individuals who were active in the morning had significantly less access to sports terrain (e.g. football fields, swimming pools) within a 1600 m radius of their residence [25]. These findings suggest that when it comes to the morningness/eveningness of PA, a number of factors, such as environmental characteristics and distance to public social cultural facilities, may have a considerable impact on the actual diurnal PA pattern throughout the day. As very limited evidence is available for young populations, elucidating what types of barriers are associated with diurnal PA preference therefore needs further investigation.

Of note, our external validly analysis showed that the summed scores in both morning (MS) and evening (ES) were significantly related to the objectively measured PA levels in the morning (6–9 AM) and evening (6–9 PM and 9 PM– 0 AM). These findings show that this novel questionnaire corresponds well with the PA behaviors in the morning and evening. Interestingly, MS or ES was not always associated with either the validated MEQ (overall preferred clock time) or an individual’s typical rise/bed time. For instance, in our data, evening PA preference was associated with morningness assessed using the MEQ (not significant). Taken together, our data suggest that our novel MEEPQ captured not the overall morningness-eveningness but the objectively measured PA. From a clinical point of view, these data suggest that individuals who prefer morning/evening do not always prefer to exercise in the at that time of day. In addition, our novel questionnaire makes it very easy to calculate the summed score. Conveniently capturing the morningness-eveningness of PA using this MEEPQ can help understand individual’s clock time regarding PA, at least in real-life situations. We believe that this questionnaire will prove clinically important when used in university and schools. Recently, Roveda et al. [26] developed an equation predicting the diurnal PA rhythm. Using the MEQ score and actigraphy, they developed a regression model to predict the acrophase in the three chronotypes (Morning-, Neither-, and Evening-types) [27]. However, their work is based on the MEQ so validation studies using the established MEQ and our novel MEEPQ should be performed in the future.

The test-retest reliability after 1 month was moderate (0.44 in the morning and 0.47 in the evening). This may be explained in part by the evidence that the chronotype is modifiable, especially by exercise. Indeed, Chtourou et al. reported that the training time-of-day was able to modify chronotype, with evening training during Ramadan shifting the MEQ scores over time [28]. Because PA may act as a behavioral modifier of the chronotype, the morningness-eveningness of PA may fluctuate and should thus be referred to as the ‘current morningness-eveningness of PA’.

This questionnaire can be useful as a new tool for increasing the PA level among young people. A number of questionnaires for assessing the mood before, during and after exercises have been developed. Previous studies have shown that morningness-eveningness of PA can influence the mood during exercise. Recently published articles reported lower ratings of perceived exertion during exercise sessions performed at their preferred time of day [7, 29]. Although the physiological mechanisms underlying these findings remain unclear, these previous articles suggest that understanding individuals’ morningness-eveningness of PA can help reduce exercise barriers thereby increase adherence of PA. How to promote PA is an important public health problem that is attracting increasing attention. The decrease in the PA levels among young Japanese individuals over the past few decades has become a social issue [30]. According to the WHO, 31.6% of adult (aged 15 years or older) were physically inactive [31]. For instance, while only 17.0% in southeast Asian were inactive, 60.2% were inactive in Japan [31]. Determining specific diurnal preferences of PA can trigger or increase motivation to establish PA habits among young individuals. The next stage will be to design PA interventions to identify which determinants can serve as vehicles of PA behavior change, as well as to specify which determinant-specific strategies are most effective in helping to produce the desired change.

### Limitations

Several limitations associated with the present study warrant mention. First, in the present study, the questionnaire was unable to distinguish the PA category. This was because we aimed to develop an easy-to-use, convenient questionnaire. PA is a form of activity that generally includes self-conscious activity (such as sport and leisure time PA) and everyday life (such as transportation and shopping). A previous study revealed that, while domain-specific PA levels were not associated with the health-related QOL among university students, the positive relationship of leisure-time, transport and domestic PA was associated with the health-related QOL [32]. Furthermore, we were unable to examine acute and chronic preferences for sports participation. Whether or not individuals with high MS/ES scores prefer to engage in sports at the corresponded time merits further investigations.

Second, it should be noted that individuals with a higher MS or ES in the MEEPQ may not be necessarily prefer exercising at the corresponding time, due perhaps to unavoidable (or compulsory) social events such as classes, part-time jobs, etc. Therefore, the objectively measured PA levels in the present study might underestimate participants’ morningness-eveningness of PA. However, our aim was to develop a convenient questionnaire. The fact that MS/ES was significantly associated with the objectively measured PA levels at the corresponding time shows that our novel MEEPQ was indeed useful for conveniently assessing the overall morningness-eveningness of PA. However, further studies will be needed in order to validate this questionnaire under stricter conditions.

Third, the findings of this study are limited mainly by the homogenous nature of the sample, as we only examined university students who were generally active (rate of participation in sports clubs was 67.5% in males and 6.3% in females; data not shown). Future studies should verify the nature of the MEEPQ in more heterogeneous young populations, including sedentary individuals and highly active individuals (such as elite athletes), to allow for greater generalization of the current findings to a broader young adult population.

Finally, because of its cross-sectional design, inferences regarding the relationship between the MEEPQ maintenance of PA over extended periods of time will need to be evaluated through intervention studies.

## Conclusions

The MEEPQ showed relatively good agreement and thus can be used for Japanese university student samples. In this novel questionnaire, three factors (physical wellness, psychological well-being and exercise barrier) contributed to the morning and evening PA preference, with the PA preference associated with physical wellness in the morning and psychological well-being in the evening. Furthermore, the summed scores were significantly related with the objectively measured PA levels in the corresponding hours. These data suggest that the MEEPQ appears to be a valid, suitable and convenient tool for assessing the diurnal PA preference in young populations. Nevertheless, further studies are warranted to evaluate the feasibility of the MEEPQ in sport-related performance and long-term adherence in other young populations.

## Supporting information

S1 Fig Correlations between the MEEPQ score, rise time, bed time and MEQ score (n = 213). Spearman's coefficient. MS, morning score; ES, evening score.
(PPTX)
