# Supplementary material for: Development and initial validation of the Morningness-Eveningness Exercise Preference Questionnaire (MEEPQ) in Japanese university students
Source: PLoS One. 2018 Jul 18;13(7):e0200870. doi: 10.1371/journal.pone.0200870 (PMC6051639; doi:10.1371/journal.pone.0200870)
Supplement: S1 Fig — Spearman's coefficient. MS, morning score; ES, evening score. (PPTX) [file pone.0200870.s001.pptx]

## Slide 1
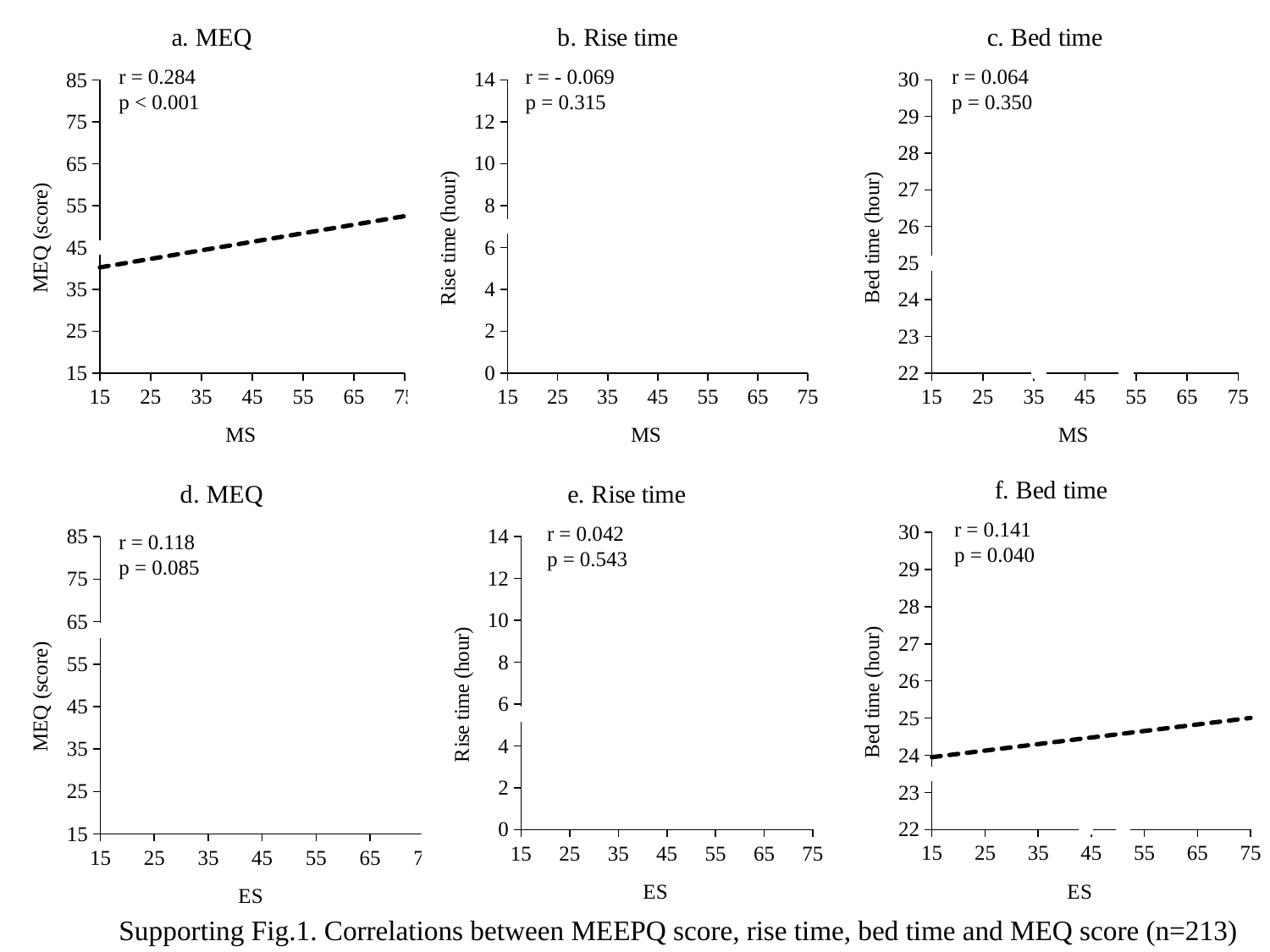

### Chart: a. MEQ
| Category | 質問紙4_朝合計得点MEEPQ |
|---|---|
### Chart: c. Bed time
| Category | 質問紙4_朝合計得点MEEPQ |
|---|---|
### Chart: b. Rise time
| Category | 質問紙4_朝合計得点MEEPQ |
|---|---|r = 0.064
p = 0.350
r = 0.284
p < 0.001
r = - 0.069
p = 0.315
### Chart: f. Bed time
| Category | 質問紙4_朝合計得点MEEPQ |
|---|---|
### Chart: d. MEQ
| Category | 質問紙4_夕合計得点MEEPQ |
|---|---|
### Chart: e. Rise time
| Category | 質問紙4_夕合計得点MEEPQ |
|---|---|r = 0.141
p = 0.040
r = 0.042
p = 0.543
r = 0.118
p = 0.085
Supporting Fig.1. Correlations between MEEPQ score, rise time, bed time and MEQ score (n=213)
